# An enhanced temperature index model for debris-covered glaciers accounting for thickness effect

**DOI:** 10.1016/j.advwatres.2016.05.001

**Published:** 2016-08

**Authors:** M. Carenzo, F. Pellicciotti, J. Mabillard, T. Reid, B.W. Brock

**Affiliations:** a Institute of Enviromental Engineering, ETH Zurich, 8093 Zurich, Switzerland; b Department of Geography, Northumbria University, NE1 8ST Newcastle, UK; c School of Geosciences, The University of Edinburgh, EH9 3JN Edinburgh, UK

**Keywords:** Debris-covered glaciers, Glacier melt modelling, Debris thickness feedback, Energy balance models, Enhanced temperature index models

## Abstract

Debris-covered glaciers are increasingly studied because it is assumed that debris cover extent and thickness could increase in a warming climate, with more regular rockfalls from the surrounding slopes and more englacial melt-out material. Debris energy-balance models have been developed to account for the melt rate enhancement/reduction due to a thin/thick debris layer, respectively. However, such models require a large amount of input data that are not often available, especially in remote mountain areas such as the Himalaya, and can be difficult to extrapolate. Due to their lower data requirements, empirical models have been used extensively in clean glacier melt modelling. For debris-covered glaciers, however, they generally simplify the debris effect by using a single melt-reduction factor which does not account for the influence of varying debris thickness on melt and prescribe a constant reduction for the entire melt across a glacier.

In this paper, we present a new temperature-index model that accounts for debris thickness in the computation of melt rates at the debris-ice interface. The model empirical parameters are optimized at the point scale for varying debris thicknesses against melt rates simulated by a physically-based debris energy balance model. The latter is validated against ablation stake readings and surface temperature measurements. Each parameter is then related to a plausible set of debris thickness values to provide a general and transferable parameterization. We develop the model on Miage Glacier, Italy, and then test its transferability on Haut Glacier d’Arolla, Switzerland.

The performance of the new debris temperature-index (DETI) model in simulating the glacier melt rate at the point scale is comparable to the one of the physically based approach, and the definition of model parameters as a function of debris thickness allows the simulation of the nonlinear relationship of melt rate to debris thickness, summarised by the Østrem curve. Its large number of parameters might be a limitation, but we show that the model is transferable in time and space to a second glacier with little loss of performance. We thus suggest that the new DETI model can be included in continuous mass balance models of debris-covered glaciers, because of its limited data requirements. As such, we expect its application to lead to an improvement in simulations of the debris-covered glacier response to climate in comparison with models that simply recalibrate empirical parameters to prescribe a constant across glacier reduction in melt.

## Highlights

• We develop a melt model for debris-covered ice accounting for debris thickness.
• The model can reproduce the melt reduction of the so-called Oestrem curve.
• The model is transferable in time over seasons and in space to a second glacier.

## 1 Introduction

Debris-covered glaciers, which are mantled in an extensive layer of debris over at least part of the ablation area, are important features of many mountainous areas of the world, from the Himalaya-Karokoram-Hindukush (HKH) region to the European Alps and North-America. Since they commonly reach lower elevations than debris-free glaciers, they are important for their contribution to water resources, and play a key role for the hydrology of high elevation catchments (Ragettli et al., 2015). Nevertheless their response to climate is not fully understood yet, which hinders a sound assessment of catchment melt and runoff, but it is clear that it differs from that of debris-free glaciers (Benn, Bolch, Hands, Gulley, Luckman, Nicholson, Quincey, Thompson, Toumi, Wiseman, 2012, Ragettli, Bolch, Pellicciotti).

The presence of a thin debris layer enhances ablation through increased absorption of shortwave radiation at the surface, compared with bare ice, and shorter vertical distance for heat conduction, while a thick cover reduces ablation as insulation dominates over increased absorption of shortwave radiation (Lejeune, Betrand, Wagnon, Morin, 2013, Nicholson, Benn, 2006, Reid, Brock, 2010). The point of divergence between melt enhancement and reduction by debris cover is termed the critical thickness. The value of the critical thickness has been shown to vary between locations depending on the debris properties and climatic setting (Reznichenko et al., 2010). The shape of the extrapolated melt rate-debris thickness relationship has often been referred to as the Østrem curve following Østrem (1959).

In general, a debris layer is assumed to reduce ablation at the glacier scale, as extensive debris cover tends to be thicker than the critical thickness (Fyffe, Reid, Brock, Kirkbride, Diolaiuti, Smiraglia, Diotri, 2014, Ragettli, Pellicciotti, Immerzeel, Miles, Petersen, Heynen, Shea, Stumm, Joshi, Shrestha, 2015). Recent remote sensing studies, however, have provided evidence of mass losses over debris-covered glaciers as large as those over debris-free glaciers (Gardelle, Berthier, Arnaud, 2012, Gardelle, Berthier, Arnaud, Kaab, 2013, Kääb, Berthier, Nuth, Gardelle, Arnaud, 2012). They have thus suggested an anomalous behavior that might be explained by the presence of supra-glacial features such as ice cliffs and lakes that develop over debris-covered glaciers and absorb heat considerably, favouring mass losses (Buri, Pellicciotti, Steiner, Miles, Immerzeel, 2016, Miles, Pellicciotti, Willis, Steiner, Buri, Arnold, 2016, Sakai, Takeuchi, Fujita, Nakawo, 2000). The evidence is limited to a very recent period and has been obtained only through remote sensing estimates of glacier mass balances, and never through numerical modelling at the glacier scale, and might therefore need further investigation. Despite this evidence at the glacier scale, however, it is clear that, at small scales, a layer of debris over ice reduces melt starting from few centimetres.

For calculations of melt rate under debris, two types of approaches have been commonly applied. On one side, physically-based energy balance (EB) models calculate the exchange of energy between the debris layer and the atmosphere on top, and ice melt at the bottom of the debris is computed as the heat transferred at the interface between ice and debris, often assuming that the ice is at melting point (Reid and Brock, 2010). This type of approach requires numerous input meteorological variables (radiative fluxes, short and longwave radiation fluxes, wind speed, air temperature, relative humidity) as well as surface variables such as surface roughness, albedo and debris water content. On the other side, at the catchment scale and in particular in data scarce regions, melt under debris has been calculated with empirical models (such as simple temperature index, TI, models) after recalibration of their parameters for debris conditions (e.g. Brown, Racoviteanu, Tarboton, Gupta, Nigro, Policelli, Habib, Tokay, Shrestha, Bajracharya, Hummel, 2014, Jouvet, Huss, Funk, Blatter, 2011, Ragettli, Pellicciotti, Bordoy, Immerzeel, 2013b, Immerzeel, Pellicciotti, Bierkens, 2013). In general, smaller values of the empirical melt parameters are used for debris than for clean ice, to reproduce the assumed average reducing effect of debris over melt. While the application of energy balance models is constrained by data availability, which are either not available in many areas or difficult to extrapolate or model, the latter approach has the disadvantage that it prescribes a constant in space reduction of melt. In reality, different melt rates are associated with different debris thickness (Fyffe et al., 2014), a fact nicely summarised in the Østrem curve, and spatial variability of debris thickness is common on debris-covered glaciers (Foster et al., 2012). This spatial variability is neglected in empirical models and can lead to erroneous simulations of total melt at the glacier scale.

In this paper, we suggest a new approach for calculations of melt rates under debris that retains the limited amount of input data typical of temperature index models but introduces a parameterisation to account for the effect of debris thickness. We build upon the enhanced temperature index model developed for calculation of melt over debris-free ice by Pellicciotti et al. (2005) and Carenzo et al. (2009) and used in numerous other applications Pellicciotti, Helbing, Rivera, Favier, Corripio, Araos, Sicard, Carenzo, 2008, Pellicciotti, Ragettli, Carenzo, McPhee, 2013, Ragettli, Cortez, McPhee, Pellicciotti, Ragettli, Pellicciotti, Immerzeel, Miles, Petersen, Heynen, Shea, Stumm, Joshi, Shrestha, 2015 and modify that model to account for varying debris thickness. We therefore suggest an approach that is intermediate between empirical methods and full energy balance models. To develop the new model we use melt rates simulated with a debris energy balance model, and calibrate the new model empirical parameters against the EB simulations. As reference, we use the debris EB (DEB) model developed by Reid and Brock (2010) using data from Miage Glacier, Italian Alps. We use the same Miage data sets also for the development of the new Debris Enhanced Temperature Index (DETI) model, and test the model developed in this way with meteorological and ablation data collected at one Automatic Weather Station (AWS) over a debris-covered section of Haut Glacier d’Arolla, Switzerland.

## 2 Study sites and data

**Fig. 1:**
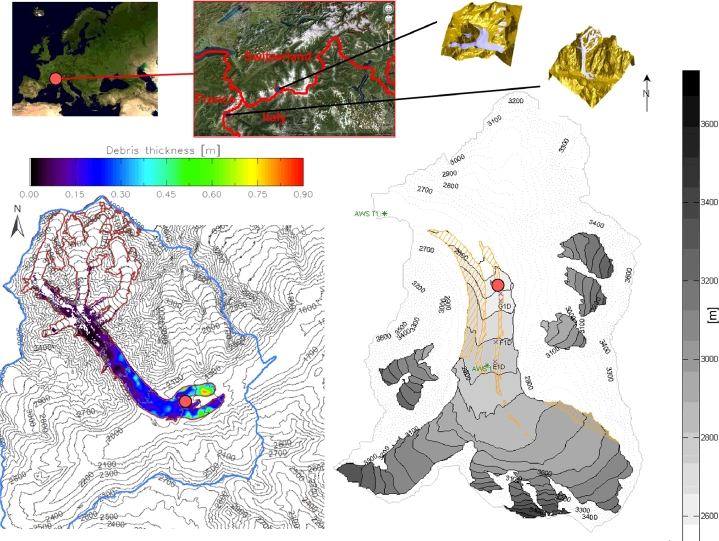
Map of Miage Glacier (left) and Haut Glacier d’Arolla (right). The map of Miage Glacier includes the debris thickness information derived by Foster et al. (2012), whereas only the debris cover extent is shown for Haut Glacier d’Arolla (orange hatched area). The red dots indicate the locations of the AWSs used in this study.

This study is undertaken on two different glaciers, Miage Glacier and Haut Glacier d’Arolla (Fig. 1). Miage Glacier is a heavily debris-covered glacier located in northwest Italy (45°47’N, 06°52’E). Haut Glacier d’Arolla, located in the southern part of Switzerland (45°59’N, 07°29’E), is mainly debris-free but is experiencing an increase of debris cover over bare ice surface (Reid et al., 2012). Brock et al. (2010) provide an extensive description of the data collected on Miage Glacier, whereas for the data from Haut Glacier d’Arolla the reader is referred to Reid et al. (2012) and Carenzo (2012).

This study is carried out at the point scale and it uses data collected at two Automatic Weather Stations (AWSs) during the 2009 and 2010 ablation seasons on Miage Glacier and Haut Glacier d’Arolla, respectively. Data collected during five additional ablation seasons (2005, 2006, 2007, 2010 and 2011) on Miage Glacier are also used to investigate the model transferability in time. A detailed description of these data sets can be found in Reid and Brock (2010). The AWS located on Miage was installed on a 23 cm debris layer, whereas debris thickness *measured at a stake close to* the AWS location in Haut Glacier d’Arolla was 6 cm and it is assumed to be the value at AWS.

For this study, we apply on Miage Glacier the same parameter set as Reid and Brock (2010). On Haut Glacier d’Arolla, in absence of site specific parameters, we use the same values as for Miage Glacier as assumed in Reid et al. (2012). Debris properties [thermal conductivity (0.94 W m^−1^ K^−1^), surface roughness (0.016 m) and albedo (0.13)] are assumed constant in time.

On Haut Glacier d’Arolla no direct observations of surface temperature are available. This variable is thus derived from the longwave radiation measurements according to Stefan Boltzmann law as:

(1) $$T_{s}={\left(\frac{OLW-((1-\epsilon )\cdot ILW)}{\epsilon \cdot \sigma }\right)}^{1/4},$$

where *ILW* and *OLW* are the incoming and outgoing longwave radiation, respectively, ϵ is the debris emissivity and *σ* is the Stefan Boltzmann constant. Surface temperature is used in model evaluation and not as model input, as the DEB model calculates surface temperature internally.

## 3 Methods

The model presented in this study is a modification of the enhanced temperature index model of Pellicciotti et al. (2005), in which melt was calculated as a sum of the full shortwave radiation balance and of a temperature dependent term. We use the same model but modify it to include the dependency of melt rates on debris thickness. The approach to derive the new model is as follows: we first run the energy balance model by Reid and Brock (2010) and evaluate it against surface temperature records at the AWSs on Miage and Haut Glacier d’Arolla. We then use it as reference to develop, calibrate and validate the new DETI model, since stake readings are too coarse a data set for univocal parameter calibration. Finally, we compare the results obtained with the new DETI model to melt simulations obtained with the ETI model with parameters recalibrated for debris conditions, to assess the performance of the new model in comparison to the more traditional empirical method of melt calculations under debris.

### 3.1 Debris Energy Balance (DEB) model

The point scale debris energy balance (DEB) model developed by Reid and Brock (2010) is used as reference for the calibration and validation of the DETI model. A detailed description of the DEB model can be found in Reid and Brock (2010). Here we only report the main model features.

The sum of energy fluxes at the surface is computed as

(2) $$Q_{I}+L_{net}\left(T_{s}\right)+Q_{H}\left(T_{s}\right)+Q_{L}\left(T_{s}\right)+Q_{R}\left(T_{s}\right)+Q_{C}\left(T_{s}\right)=0$$

where *Q_I_* is net shortwave radiation, *L_net_* is net longwave radiation, *Q_H_* and *Q_L_* are sensible and latent heat fluxes, *Q_R_* is heat flux supplied by rain and *Q_C_* is conductive heat flux into the debris layer. Debris surface temperature *T_s_* is assumed to change at each time step (1 hour) and Eq. 2 is solved for *T_s_* using a numerical Newton-Raphson method. The Crank-Nicholson scheme is used to compute the heat conduction through the debris layers (1 cm thick). The boundary conditions are represented by the newly-calculated *T_s_* and the temperature at the debris-ice interface, which is assumed equal to 0 °C. The debris albedo is set to a constant value of 0.13 following Reid and Brock (2010), and all other model parameters (surface roughness, conductivity of the debris, etc) are also taken from that study.

The melt rate is derived from the conductive heat flux to the ice (*Q*_*C*, bottom_), calculated by means of the temperature gradient (*Δt*) between the lowest debris layer and the ice underneath:

(3) $$M=\frac{Q_{C,\text{bottom}}\Delta t}{\rho_{w}L_{f}},$$

where *ρ_w_* is the density of water and *L_f_* is the latent heat of fusion for water.

The DEB point model outputs were validated against surface temperature measurements at Miage Glacier during the 2005, 2006 and 2007 ablation seasons (see Reid and Brock (2010) for details).

The DEB model cannot replicate the reduction in melt rate for very thin debris that is suggested by the Østrem curve, for reasons discussed extensively in Reid and Brock (2010). While it is clear that the melt rate increases for thin debris layers, no EB model at yet has provided evidence that it reaches a maximum and then decreases towards the bare-ice melt rate as the debris thickness tends towards zero. This effect was obtained only by Reid and Brock (2010) using a patchy debris scheme, and more recently by Evatt et al. (2015) by incorporating debris layer air flow. These promising additional schemes need testing and more experimental evidence, and for the development of the DETI model we thus use the original DEB model of Reid and Brock (2010). As a result, the DETI model will suffer from the same limitations as the DEB model for thin debris, and will be used only to study the reducing effect of thick debris on melt rates.

### 3.2 Debris Enhanced Temperature-Index (DETI) model

Temperature index or degree-day models are based on empirical relationships between air temperature and melt rate (Hock, 2005, Pellicciotti, Brock, Strasser, Burlando, Funk, Corripio, 2005). The main advantage of such empirical models is the lower data requirement in comparison to physically based energy balance models. The enhanced temperature index model (ETI model) developed by Pellicciotti et al. (2005) is an intermediate step between an empirical and an energy balance model. In addition to the air temperature term, the ETI model includes a shortwave radiation term which incorporates incoming solar radiation and albedo. Hourly melt rates ($\mathrm{mm}\;w.e.h^{-1}$) are computed as

(4) $$\begin{array}{c} M=\left\{ \begin{array}{cc} TF\;\cdot \;T\;+\;SRF\;\cdot \;(1-\;\alpha \;)\;\cdot \;I & T\;>\;T_{T}\\ 0 & T\;\leq \;T_{T} \end{array}\right. \end{array}$$

where *T* is air temperature (°C), *α* is albedo (-), *I* is incoming shortwave radiation (W m^−2^) and the two empirical factors *TF* and *SRF* are the temperature factor (mm h^−1^°C^−1^) and the shortwave radiation factor (m^2^ mm W^−1^ h^−1^), respectively. *T_T_* is an additional parameter and corresponds to the threshold temperature above which melt is assumed to occur.

In previous works, *TF* and *SRF* were adjusted for melt under debris and recalibrated against stakes readings or EB simulations (Ragettli et al., 2013b). Similar approaches have been adopted by e.g. Immerzeel et al. (2012). However, the accuracy and transferability of this approach is limited by the lack of a term representative of the debris thickness feedback. The parameter calibration can lead to an improvement in the melt rate computation for a specific debris thickness value, but it can not reproduce the behaviour suggested by Østrem (1959).

For this reason, we propose a new empirical approach accounting for the debris thickness feedback in the melt rate computation. The Debris Enhanced Temperature-Index (DETI) model calculates hourly melt rates ($\mathrm{mm}\;w.e.h^{-1}$) as

(5) $$\begin{array}{c} M=\left\{ \begin{array}{cc} TF\;\cdot \;T\left(i-lag_{T}\right)\;+\;SRF\;\cdot \;\left(1-\;\alpha \;\right)\;\cdot \;I\;\left(i-\;lag_{I}\right) & T\;>\;T_{T}\\ 0 & T\;\leq \;T_{T} \end{array}\right. \end{array}$$

where *i* is the timestep (h), and *lag_T_* and *lag_I_* are lag parameters accounting for the energy transfer through the debris layers.

*T* is temperature (°C), *α* is albedo (-), *I* is incoming shortwave radiation (W m^−2^). To find the *lag_T_, lag_I_, TF* and *SRF* values, we optimize them at the point scale for several debris thicknesses against melt rates simulated by the DEB model. We then parameterise each empirical factor in Eq. 5 as a function of debris thickness and additional empirical parameters (*lag*_1_, *lag*_2_, *TF*_1_, *TF*_2_, *SRF*_1_ and *SRF*_2_) as:

(6) $$lag=lag_{T}=lag_{I}=lag_{1}\cdot d+lag_{2}$$

(7) $$TF=TF_{1}\cdot d^{TF_{2}}$$

(8) $$SRF=SRF_{1}\cdot e^{SRF_{2}\cdot d}$$

where *d* is the debris thickness (m). The lag parameter accounts for the delay in melt caused by the transmission of the energy absorbed at the debris surface to the ice, and it is clearly dependent on debris thickness, with thicker debris increasing the delay. The *TF* and *SRF*, which multiply air temperature and the shortwave radiation balance Eq. 5) represent the reduction in melt rates associated with thicker debris. The model development (including the functional form of (5), (6), (7) and (8) and the results of the parameter optimization are described in Section 4.3.

## 4 Results and discussion

### 4.1 DEB validation

**Fig. 2:**
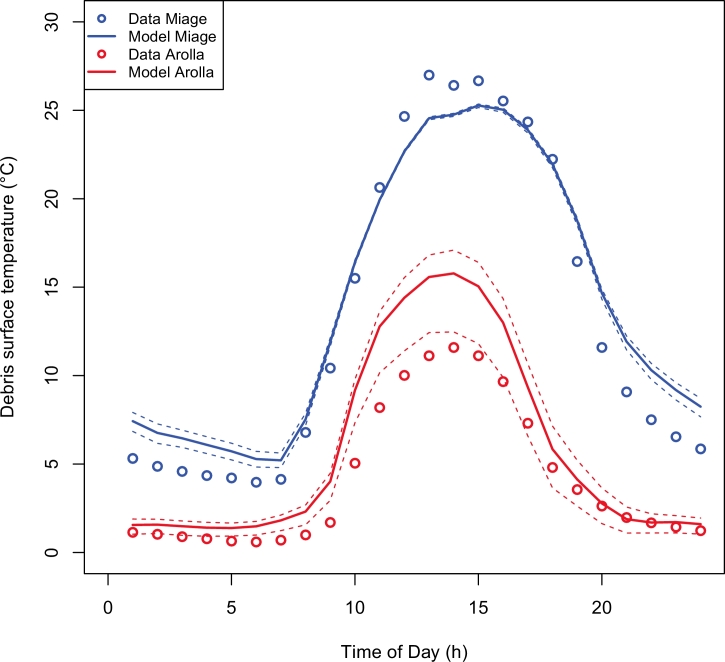
Mean daily cycles of modelled and measured debris surface temperature on Miage Glacier during the 2009 ablation season (blue) and on Haut Glacier d’Arolla during the 2010 ablation season (red). Uncertainty ranges obtained by running the DEB model with debris thickness values changed by ±3 cm from the reference debris thickness of 23 and 6 cm (for Miage Glacier and Haut Glacier d’Arolla, respectively), are included for both glaciers.

Reid and Brock (2010) evaluated the DEB model at the debris-covered Miage Glacier for the 2005, 2006 and 2007 ablation seasons. Therefore in this paper we only validate the results of the DEB model for the new ablation season at Miage Glacier (2009 ablation season), and the new study site, Haut Glacier d’Arolla (2010 ablation season). The model is validated by comparing the mean daily cycles of measured and modelled debris surface temperature, following Reid and Brock (2010) (Fig. 2). Measurements of surface temperature from radiometers, obtained from records of outgoing longwave radiation by inverting Stefan-Boltzman relationship (Eq. 1), can have significant uncertainty due to sample bias on a highly variable field of surface temperature. We used a CNR1 net radiometer that was installed at 2m above the surface. Thus, 99% of the input to the lower sensor came from a circular area with a radius of 20 m (Campbell Scientific Instruction Manual). In this area, debris thickness was not constant at the value of 6 cm measured at the stake in proximity of the AWS, but varied significantly so that the field of view of the radiometer very likely incorporated areas of varying debris thickness, and of thinner debris in particular. To account for this, we compare the observations to the modelled values with 6 cm thickness as well as with those obtained by varying by ±3 cm around 6 cm, which should represent some of the variations observed in the debris thickness in the area. The effect of varying debris thickness on the variability of surface temperature is particularly strong for thin debris, so that we expect the heterogeneity of the debris layer to be more important at Haut Glacier d’Arolla than at Miage Glacier.

The Nash and Sutcliffe model efficiency criterion (*NSE*) used to express the model performance shows a very good fit between observations and modelled outputs at Miage Glacier (*NSE* = 0.913 for the original setting with d=23 cm), also considering the uncertainty in the measurements of surface temperature. The Root Mean Square Error, *RMSE*, is 2.88 °C, and correlation coefficient (*r*) is 0.966. At Haut Glacier d’Arolla, agreement between observations and model outputs is less good for the simulations with 6 cm thickness (*NSE* = 0.718, *RMSE* = 2.66 °C, *r* = 0.971), but improves considerably for 3 cm (*NSE* = 0.910, *RMSE* = 1.50 °C, *r* = 0.996), suggesting that the debris thickness around the AWS is variable and likely thinner than 6cm, which would explain the lower surface temperature. Another reason for the lower performance of the DEB model at Haut Glacier d’Arolla could be due to the lack of direct observations of debris properties, with values of surface roughness and debris thermal conductivity taken from Miage. Results of a model sensitivity analysis (not shown here) have shown however that these would not explain the observed discrepancy.

### 4.2 Melt rates and energy transfer through the debris layers: DEB model outputs

**Fig. 3:**
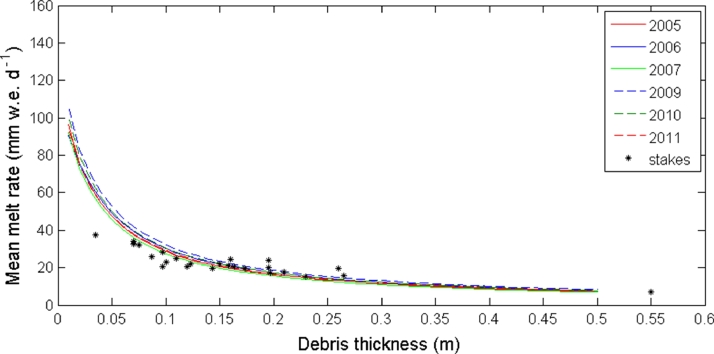
Mean daily melt rates computed with the DEB model assuming debris thickness values varying from 0.1 to 50 cm for the six ablation seasons investigated at Miage Glacier. The ablation stake readings from the 2005 ablation season are also included.

The Østrem curve is built by running the DEB model using the meteorological forcing at the AWS on Miage Glacier during the six ablation seasons and varying the debris thickness from 0.1 to 50 cm (Fig. 3). The ablation stake readings during the 2005 ablation season are also included in Fig. 3. The results show a relatively consistent behaviour and similar melt rate values over the six years investigated. Thick debris layers produce low melt, whereas melt rates increase when debris becomes thinner, following the general prescribed behaviour. Differences among seasons are small compared to the effect of thickness, suggesting that the meteorological forcing is less important to melt variations than debris thickness, particularly in the case of thick debris layers.

The Østrem curve obtained by forcing an EB model with meteorological variables collected at one site and varying debris thickness is a theoretical exercise, as meteorological variables such as air temperature or the atmospheric boundary layer can vary with thickness (Reid and Brock, 2010). By assuming the same time series of atmospheric forcing, such additional debris effects at the interface with the atmosphere are not taken into account. Moreover, very thin debris cover that dramatically enhances melt is very unlikely to be found over large areas. Thin debris is generally spread out and patchy, with some areas exposed to bare ice that reduce the overall effective ablation of the area. Thus, the behavior of the simulated curve (Fig. 3) for very thin debris should be closer to the bare ice melt rate, as suggested by the original Østrem curve.

**Fig. 4:**
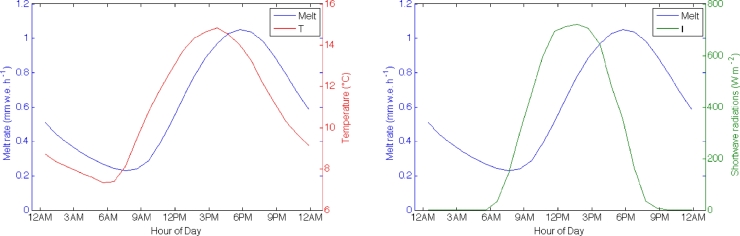
Mean daily cycles of melt rate simulated by the DEB model at the AWS on Miage Glacier during the 2005 ablation season with (left) air temperature and (right) incoming shortwave radiation.

**Fig. 5:**
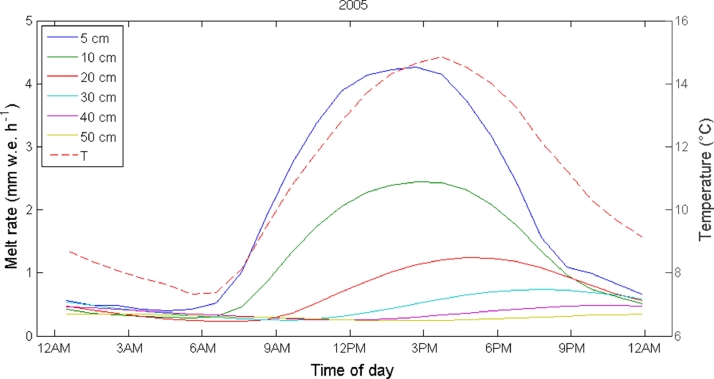
Mean daily cycles of melt computed by the DEB model for different debris thicknesses (solid lines) compared to the mean daily cycle of air temperature (*T*) observed at the AWS on Miage Glacier (dashed line) during the 2005 ablation season.

**Fig. 6:**
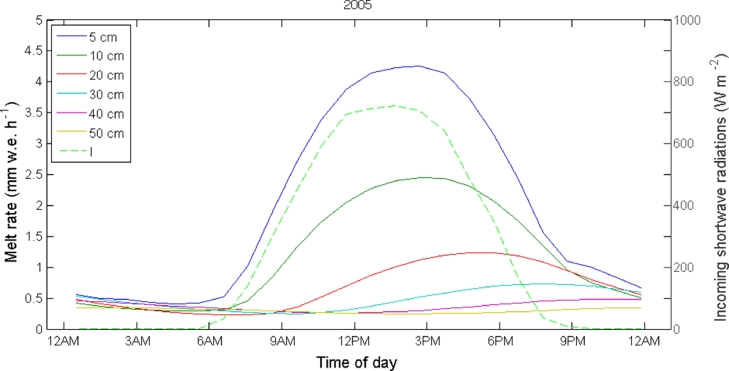
Mean daily cycles of melt computed by the DEB model for different debris thicknesses (solid lines) compared to mean daily cycle of incoming shortwave radiation (*I*) observed at the AWS on Miage Glacier (dashed line) during the 2005 ablation season.

The relationship between melt rates and the main atmospheric forcing is investigated by comparing the mean daily cycle of air temperature and incoming shortwave radiation to the melt rate cycle simulated by the DEB model (Fig. 4). On Miage Glacier a lag between air temperature and incoming shortwave radiation with melt is evident (Fig. 4). In particular, a clear shift between the peaks of the two cycles is visible in Fig. 4. The lag represents the time needed for the energy transfer through the debris layer, and is proportional to the debris thickness (Figs. 5 and 6), in agreement with Fourier law of heat conduction. A higher lag corresponds to a thicker debris layer (Figs. 5 and 6).

The two main aspects emerging from analysis of the DEB simulations and discussed in this section are thus: 1) Melt rate decreases with the increase of debris thickness (Fig. 3), and 2) the lag between the peaks of the daily cycles of air temperature and shortwave radiation versus melt rate increases with the increase of debris thickness. These are the two features that we attempt to incorporate into the DETI model.

### 4.3 DETI development

Table 1 lists the recalibrated DETI parameters (*lag_T_, lag_I_, TF, SRF*) obtained for each debris thickness and the corresponding statistical performance. The model performance as represented by the *NSE* is in general very high. It is lower for the two highest values of debris thickness, going from 0.937 at 0.3 m to 0.875 at 0.4 m and 0.624 at 0.50 m. This is due to the fact that the NSE is lower for low numerical values of the target variables, and low values of melt rates are typical of higher debris thickness. The NSE is a normalised measure that compares the mean square error generated by a model simulation to the variance of the observed variable time series, and is thus higher for cases where the variability in the time series of the target variable is higher (Schaefli and Gupta, 2007). The low NSE values corresponding to the two thickest debris do not necessary indicate a lower performance, as pointed by the low values of the RMSE corresponding to these two cases (Table 1).

Lag parameters for air temperature (*lag_T_*) and incoming shortwave radiation (*lag_I_*) assume generally the same value (Table 1). The small differences are due to the fact that the diurnal cycle of air temperature is slightly delayed compared to the incoming shortwave radiation one.

**Fig. 7:**
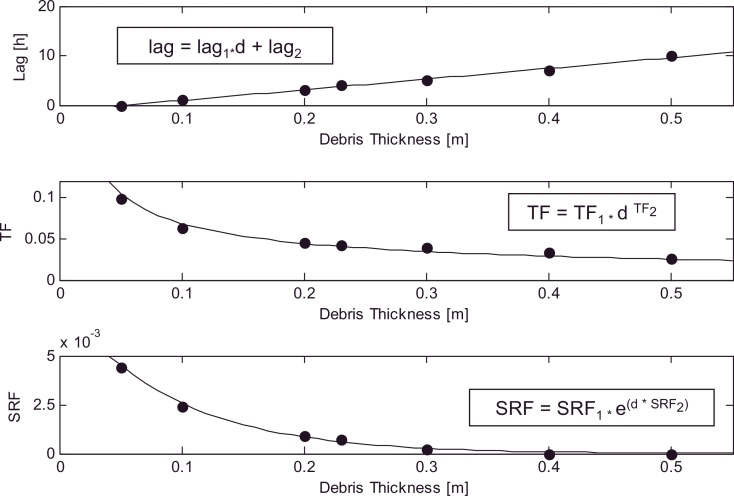
Recalibrated *lag* (top), *TF* (center) and *SRF* (bottom) against debris thickness values [*d*] at the AWS on Miage Glacier in 2005. The equations describing the best model fit are also included in the three plots.

In light of the results shown in Table 1 and in order to reduce the number of parameters, *lag_T_* and *lag_I_* are condensed in a single term (*lag*). This assumption leads only to a slight reduction of the DETI model performance (results not shown), which is considered acceptable in view of the gained computational benefits. *lag, TF* and *SRF* are then expressed as a function of debris thickness. The debris thickness feedback implies that *lag, TF* and *SRF* are variables. Their relationship with debris thickness is investigated in Fig. 7. *lag* shows a remarkably linear behaviour with debris thickness (Fig. 7) and is approximated with a linear regression with slope *lag*_1_ and intercept *lag*_2_. Two parameters are thus included in the DETI model and the model calibration leads to *lag*_1_ = 21.54 and *lag*_2_ = –1.193.

*TF* and *SRF* also decrease with debris thickness due to the decrease of melt associated with thicker debris layers. However, their behaviour is not linear (Fig. 7) and we use a different function to describe the two relationships. This choice is justified by the different effect on melt rates and relation to debris thickness of the the two variables and associated energy contributions. Incoming shortwave radiation has a daily cycle (0 at night) and energy gained during the day is given back to the atmosphere at night enabling a decoupling of the debris surface energy balance from the ice-debris interface for thick debris. On the other hand, so long as temperature is positive it can always contribute energy to the debris-ice interface, thus justifying different functional forms. We tried different functions and used those with the best fit to the data. As a result, *TF* varies with debris thickness assuming a power law, whereas an exponential decrease is adopted for *SRF*. The model calibration leads to *TF*_1_ = 0.016 , *TF*_2_ = –0.621, *SRF*_1_ = 0.0079, and *SRF*_2_ = –11.21.

**Fig. 8:**
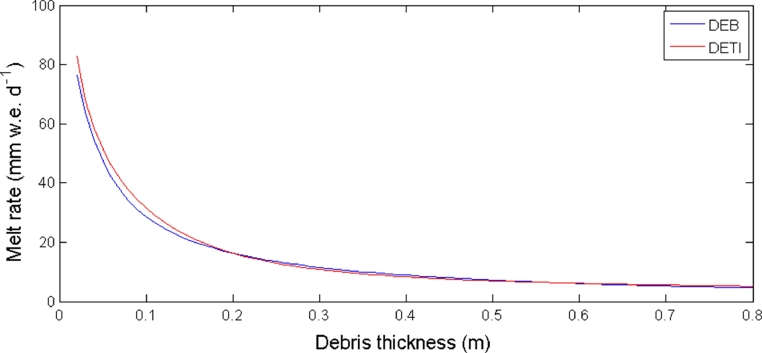
Østrem curves simulated by the DETI and the DEB model at the AWS on Miage Glacier. Meteorological data collected at the AWS during the 2005 ablation season are used as input to the model simulations.

Fig. 8 shows the comparison between the Østrem curve obtained by the DETI model and the one simulated by the DEB model on Miage Glacier during the 2005 ablation season. The two curves present a similar behaviour. Higher discrepancies occur for thin debris layers, when the DETI model slightly overestimates the mean daily melt rate. The models do not replicate the reduction in melt rate for thin debris above the critical thickness.

**Fig. 9:**
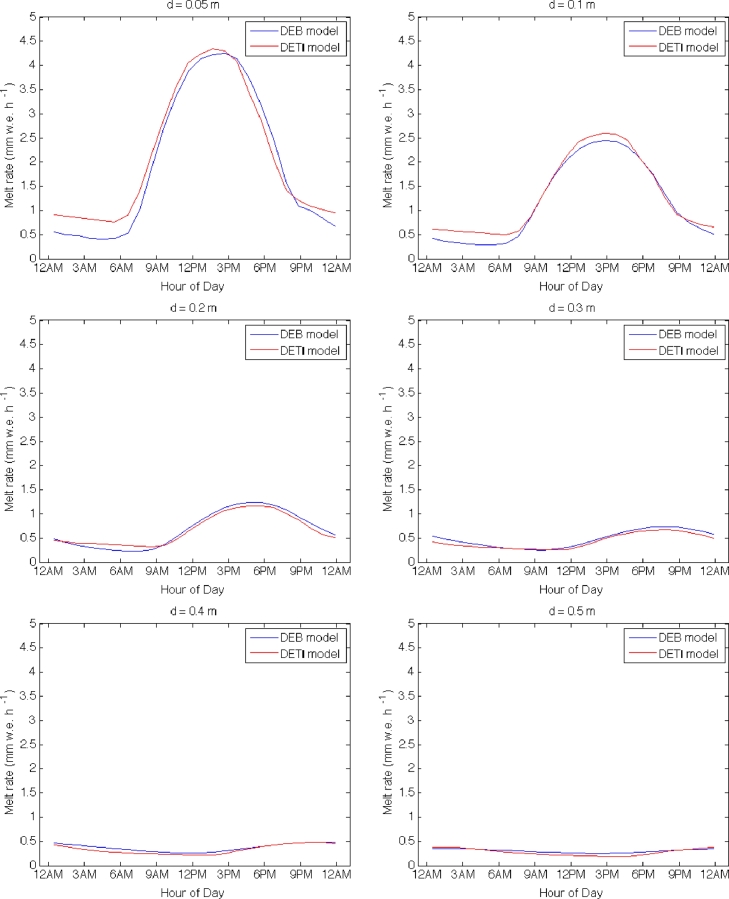
Mean daily cycles of melt rate simulated at the AWS on Miage Glacier by the DETI and the DEB model assuming debris thickness values varying from 0.05 to 0.5 m. Meteorological data collected at the AWS during the 2005 ablation season are used as input to the model.

In order to investigate further the DETI model performance, the mean daily cycle of melt rate simulated by the new empirical debris model is compared to the one obtained using the DEB model for varying thicknesses (Fig. 9). For thin debris layers (0.05 m and 0.1 m), the DETI model tends to slightly overestimate the melt rates, especially during the night. For thicker debris, the two mean daily cycles are very close. Overall, the DETI model performance is high and the model can reproduce the decrease of melt caused by the increase of debris thickness. The *lag* factor accounting for the energy transfer through the debris layer produces a substantial improvement in comparison to the results obtained with a more classical empirical model.

### 4.4 DETI versus ETI recalibrated for debris conditions

The increase in model performance obtained with the new model is assessed by comparing it to results from the ETI model calibrated for debris conditions at the AWS on Miage Glacier. Both models are compared to the DEB model outputs on Miage Glacier during the 2005 ablation season, which was the season that allowed the best validation because of the numerous ablation stake readings. The ETI model is also calibrated against hourly melt rates computed by the DEB model.

**Fig. 10:**
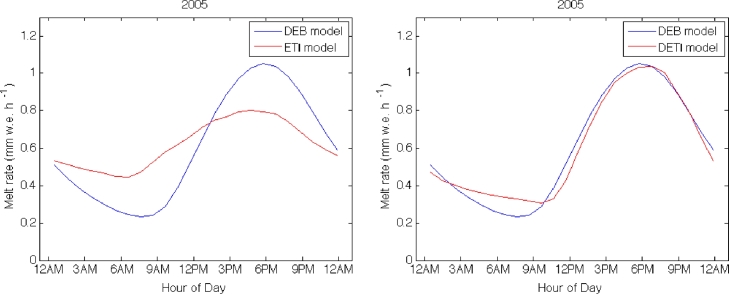
Mean daily cycles of (left) ETI model’s melt without lag parameters and (right) DETI model’s melt with the two lag parameters compared to DEB model at Miage Glacier during the 2005 ablation season.

Despite the parameter recalibration, the ETI model is not able to correctly reproduce the mean daily cycle of melt rate, as it overestimates low melt rates and underestimates high melt rates (Fig. 10). A sum of the two errors might result in daily melt rates similar to the observed ones, but these result from compensation of errors and not accurate simulations. The DETI approach, on the other side, can clearly reproduce the reference mean daily cycle of melt rate (Fig. 10). Some discrepancies occur for the low melt rates during the nighttime and at the beginning of the day, but the increase in performance over the ETI is signifcant. Thus, despite being characterized by a higher number of parameters (six in total), the new formulation seems more appropriate for calculations of melt rates under debris.

### 4.5 DETI model transferability in time

The model transferability in time is assessed by applying the DETI model to five other ablation seasons, namely 2006, 2007, 2009, 2010 and 2011, on Miage Glacier. The parameter set calibrated for the 2005 ablation season (discussed in Section 4.3) is transferred as such to the other five seasons. Table 2 summarizes the Nash and Sutcliffe efficiency criteria obtained by comparing the hourly melt rates simulated by the DETI model to those computed by the DEB model. As observed in 2005, the DETI model performance is good for debris thickness ranging from 0.05 m to 0.40 m, but the *NSE* becomes lower than 0.7 for debris thickness equal to 0.5 m because of the lower actual numerical values of melt (Criss, Winston, 2008, Krause, Boyle, Baese, 2005, Legates, McCabe, 1999). The RMSE values however indicate that the actual difference between model and observations is low. In general, the agreement tends to decrease with increasing debris thickness, but this error is of lesser importance since for these debris thicknesses melt is very low.

A lower model performance is obtained during the 2009 ablation season for debris thickness equal to 0.5 m. The 2009 summer was a particularly warm season.

### 4.6 DETI model transferability in space

**Fig. 11:**
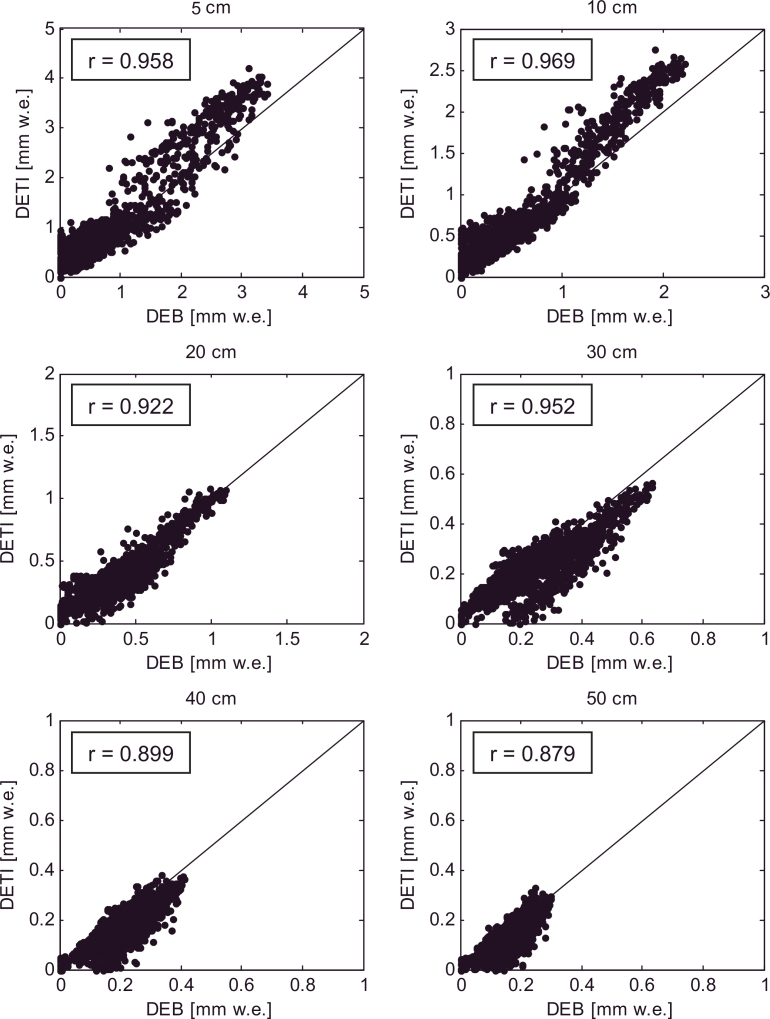
Hourly melt rates simulated by the DEB model against those computed by the DETI model at Haut Glacier d’Arolla during the 2010 ablation season. Different debris thickness values are tested, varying from 0.05 to 0.5 m. The correlation coefficient (r) is also included in order to evaluate the DETI model performance.

**Fig. 12:**
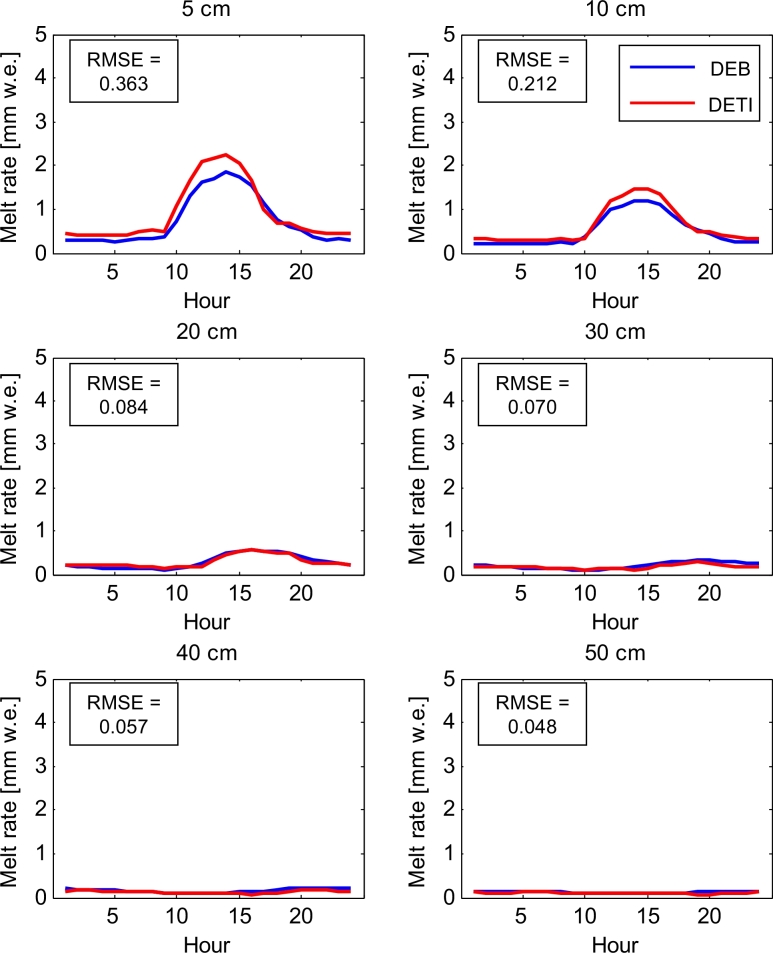
Mean daily cycles of melt rate simulated by the DETI and the DEB model assuming debris thickness values varying from 0.05 to 0.5 m. Meteorological data collected at Haut Glacier d’Arolla during the 2010 ablation season are used as input to the model simulations. The Root Mean Square Errors (*RMSE*) are also indicated to express the agreement between the hourly melt rates simulated by the DETI and the DEB model.

The DETI model transferability in space is evaluated in terms of scatterplots of hourly melt rates (Fig. 11) and mean daily cycles of melt rate (Fig. 12) at the AWS on Haut Glacier d’Arolla in 2010. Table 2 shows the NSE and RMSE calculated comparing the DETI outputs against the DEB simulations. Values of the NSE are of the same magnitude as those for Miage, except for the thinner debris layers, for which the performance is slightly lower in Arolla (but well above 0.8) (Table 2). On the other hand, the RMSEs (ranging from 0.36 °C to 0.05 °C) are in general lower in Arolla than in Miage, suggesting smaller absolute differences between the two models. It is difficult to explain the lower NSE associated with thinner debris (and evident also in Fig. 12), but we notice that the same model features (overestimation of melt rates in the first half of the day for thin debris, Fig. 12) are evident in the Miage simulations (Fig. 9), thus suggesting a consistency in model behaviour. A possible explanation for the overestimation of melt during the day for thin debris might be found in the values of the curve fitted to the optimised parameters (Fig. 7), which slightly overestimates both *TF* and *SRF* for *d* ≤ 10 cm (Fig. 7). Higher parameters would result in higher melt simulations when both *T_s_* and *I* are high, i.e. during the day hours (Eq. 5). Another possible reason for the overestimation of melt rates during the early morning and peak hours could be that the model parameters are constant over the day, while the energy fluxes are highly variable (e.g. Reid et al., 2012). While the variability of the shortwave radiation flux is explicitly included in the melt equation (Eq. 5), the diurnal changes of all other fluxes are lumped together in one temperature-dependent term where a constant *TF* multiplies air temperature (Eq. 5). The DETI model lacks an explicit representation of the strongly varying sensible heat fluxes (negative both during the day and night, but strongly during the warm hours of the day), and thus misses a negative term during the day that cannot be accounted for entirely by the calibrated *TF* as this lumps together also all other temperature dependent fluxes. This could justify the overestimation of melt rates during the day, but it is not clear why this effect would be evident for thin debris only.

The correlation coefficients (*r*) in Fig. 11, ranging from 0.969 to 0.879, also suggest good agreement between the hourly melt rates simulated by the DEB model and those modelled by the DETI, for debris thicknesses varying from 0.05 to 0.5 m, and confirm the overestimation of high melt rates for thin debris apparent in Fig. 12.

Overall, the DETI model performance at the validation site of Haut Glacier d’Arolla seems comparable to that at Miage Glacier, thus supporting the model transferability in space, at least for sites in the same broad climatic and geographic setting. The agreement between the model outputs obtained with the new empirical approach and those simulated by the reference DEB model thus remains good also when no parameter recalibration is conducted. However, the robustness of the new empirical parameters should be tested at other sites and related to debris properties, which can differ substantially for different materials and climatic conditions. As indicated above, a limitation of the model might be evident when the the energy fluxes that are represented in the lumped temperature-dependent term (*TF* · *T*) have different signs, or opposite patterns during the day or the season. These cannot likely be captured by a simplified term where the temporal variability is prescribed only by the variation of air temperature (since the *TF*, while varying with debris thickness, is constant in time). In such cases, a more physically based DEB model might be preferred. Locations with high debris moisture content (such as the debris-covered glaciers of the Himalaya during monsoon) might also not be appropriate for the application of the model without recalibration, because its parameters were calibrated for the relatively dry conditions of ablation seasons in the European Alps, where the latent heat flux is of minor significance (Brock, Mihalcea, Kirkbride, Diolaiuti, Cutler, Smiraglia, 2010, Reid, Brock, 2010).

## 5 Conclusions

In this paper, we present a new temperature-index model accounting for the debris thickness feedback in the computation of melt rates at the debris-ice interface. The model empirical parameters (temperature factor, shortwave radiation factor, and lag factor accounting for the energy transfer through the debris layer) are expressed as a function of debris thickness and optimized at the point scale for varying debris thicknesses against melt rates simulated by a physically-based debris energy balance model. The latter is validated against ablation stake readings and surface temperature measurements. Each parameter is then related to a plausible set of debris thickness values to provide a general and transferable parameterization.

We compare this approach to a simple ETI model with empirical parameters recalibrated for debris conditions. This model is not able to reproduce correctly the mean daily cycle of melt, severely underestimating the higher melt rates and overestimating the lower ones. The introduction of the lag parameter in the DETI model, by accounting for the time taken for heat transfer through debris, leads to a significant improvement in the model performance.

The performance of the new DETI model in simulating the glacier melt rate at the point scale is comparable to the one of the physically based DEB model, thanks to the definition of model parameters as a function of debris thickness. The model simulates the descending limb of the Østrem curve, whereas is not able to reproduce the melt enhancement at very thin debris thicknesses, a limitation that it shares with the original DEBI model. Both models could only be applied to thin debris using a patchy debris scheme as in Reid and Brock (2010), or by including evaporative fluxes within the debris layer, as in Evatt et al. (2015), which however is beyond the scope of this paper, but it surely should be investigated in future work.

The drawback of this approach is that it requires numerous empirical parameters that need calibration. We have shown however that they seem to be relatively stable in time at the same site and transferable in space from Miage Glacier to Haut Glacier d’Arolla in Switzerland. The two sites are in the same broad geographic and climatic setting of the European Alps, at a relative close distance and this transferability in space should thus be further investigated at other sites, both in the same region (e.g. at higher elevations) and in distinct mountainous areas such as the Andes or Alps. This task might be difficult due to lack of observations of both meteorological and surface variables as well as ablation rates from debris-covered sites, but it seems imperative to strengthen the model physical basis.

Application of the new DETI model requires estimates of debris thickness and its variability in space over glaciers, something that has been lacking due to the difficulties of direct measurements in the fields and lack of calculation methods. Recently, however, progress has been made in estimating debris thickness from satellite thermal imagery (Foster, Brock, Cutler, Diotri, 2012, Rounce, McKinney, 2014, Schauwecker, Rohrer, Huggel, Kulkarni, Ramanathan, Salzmann, Stoffel, Brock, 2015, Zhang, Fujita, Liu, Liu, Nuimura, 2011). The methods suggested are based on the inversion of the energy balance at the debris surface and knowledge of surface temperature from the satellite thermal imagery, thus solving for debris thickness as only unknown, if the input meteorological forcing to the site is known. The main uncertainty in these approaches to date is related to the non-linear profile of temperature within the debris, which causes different images to result in different thicknesses for the same site. Clear progress however has been made (Rounce, McKinney, 2014, Schauwecker, Rohrer, Huggel, Kulkarni, Ramanathan, Salzmann, Stoffel, Brock, 2015) from the first attempts (Foster, Brock, Cutler, Diotri, 2012, Zhang, Fujita, Liu, Liu, Nuimura, 2011), so that there is potential that accurate maps of debris thickness can be obtained in the near future. Combination of debris thickness distribution derived from satellite data and the DETI model could thus be applied to remote glaciers to provide improved estimates of melt in comparison to previous first order approximations calculated assuming constant thickness. Its main advantage is its limited data requirement, which makes it a novel approach that can be included in continuous mass balance models of debris-covered glaciers for long term past and future simulations.

## Figures and Tables

**Table 1:** DETI model parameters (*lag_T_, lag_I_, TF, SRF*) calibrated against hourly melt rates computed by the DEB model for debris thickness between 5 and 50 cm. The Nash and Sutcliffe model efficiency criterion (*NSE*) and Root Mean Square Error (*RMSE*) are also indicated.

Debris thickness	*lag_T_*	*lag_I_*	TF	SRF	NSE	RMSE
(m)	(h)	(h)	(mm h^−1^°C^−1^)	(m^2^ mm W^−1^ h^−1^)		°C
0.05	0	0	0.0984	0.0044	0.910	0.55
0.1	0	1	0.0660	0.0023	0.927	0.31
0.2	3	3	0.0456	0.0009	0.932	0.10
0.23	3	4	0.0438	0.0006	0.935	0.09
0.3	5	5	0.0392	0.0002	0.937	0.05
0.4	7	7	0.0334	0.0001	0.875	0.05
0.5	10	11	0.0265	0	0.624	0.05

**Table 2:** Nash and Sutcliffe Efficiency Criterion (*NSE*) and Root Mean Square Error (*RMSE*) used to assess the agreement between the DETI and DEB model hourly melt rates [mm w.e. h^−1^]. Melt rates are calculated at the AWS on Miage Glacier during the the six ablation seasons investigated in this study and at the AWS on Haut Glacier d’Arolla during the 2010 ablation season.

	Miage		Miage		Miage		Miage		Miage		Miage		Arolla	
d	2005		2006		2007		2009		2010		2011		2010	
(cm)	*NSE*	*RMSE*	*NSE*	*RMSE*	*NSE*	*RMSE*	*NSE*	*RMSE*	*NSE*	*RMSE*	*NSE*	*RMSE*	*NSE*	*RMSE*
0.05	0.906	0.56	0.924	0.52	0.901	0.54	0.896	0.58	0.906	0.57	0.904	0.52	0.830	0.36
0.1	0.915	0.32	0.933	0.30	0.899	0.34	0.908	0.34	0.910	0.35	0.907	0.33	0.852	0.21
0.2	0.928	0.11	0.931	0.10	0.928	0.11	0.920	0.12	0.925	0.11	0.920	0.11	0.906	0.08
0.3	0.886	0.05	0.872	0.06	0.881	0.06	0.864	0.06	0.880	0.05	0.882	0.05	0.816	0.07
0.4	0.781	0.05	0.778	0.05	0.777	0.05	0.708	0.06	0.791	0.04	0.798	0.05	0.748	0.06
0.5	0.568	0.05	0.638	0.05	0.589	0.05	0.334	0.06	0.651	0.05	0.629	0.05	0.702	0.05
